# Effect of Methyl Jasmonate on the Growth and Biosynthesis of C13- and C14-Hydroxylated Taxoids in the Cell Culture of Yew (*Taxus wallichiana* Zucc.) of Different Ages

**DOI:** 10.3390/biom13060969

**Published:** 2023-06-09

**Authors:** Elena Demidova, Elena Globa, Andrey Klushin, Dmitry Kochkin, Alexander Nosov

**Affiliations:** 1 K.A. Timiryazev Institute of Plant Physiology of Russian Academy of Sciences, Botanicheskaya 35, 127276 Moscow, Russia; 2 Biology Faculty, M.V. Lomonosov Moscow State University, 119234 Moscow, Russia

**Keywords:** plant cell culture, elicitation, paclitaxel, taxuyunnanine C, yunnanxane, sinenxane, plant secondary metabolism

## Abstract

The effects of methyl jasmonate (MeJ) on growth and taxoid formation in the cell culture of *Taxus wallichiana* were investigated to elucidate the specifics of phytohormone action in dedifferentiated plant cells in vitro. The characteristics of the same suspension cell culture were compared in 2017 (the «young» culture) and in 2022 (the «old» culture)—1.5 or 6 years after culture induction, respectively. MeJ (100 µM) is added to the cell suspension at the end of the exponential growth phase. Cell culture demonstrated good growth (dry weight accumulation 10–18 g/L, specific growth rate µ = 0.15–0.35 day^−1^) regardless of its «age», cultivation system, and MeJ addition. UPLC-ESI-MS analysis revealed the presence of C14-hydroxylated taxoids (yunnanxane, taxuyunnanine C, sinenxane C, and sinenxane B) in the cell biomass. The content of C14-OH taxoids increased from 0.2–1.6 mg/gDW in «young» culture to 0.6–10.1 mg/gDW in «old» culture. Yunnanxane was the main compound in «young» culture, while sinenxane C predominated in «old» culture. Without elicitation, small amounts of C13-OH taxoids (<0.05 mg/gDW) were found only in «young» cultures. MeJ addition to «young» culture had no effect on the content of C14-OH taxoids but caused a 10-fold increase in C13-OH taxoid production (up to 0.12–0.19 mg/gDW, comparable to the bark of yew trees). By contrast, MeJ added to «old» culture was not beneficial for the production of C13-OH taxoids but notably increased the content of C14-OH taxoids (1.5–2.0 times in flasks and 5–8 times in bioreactors). These findings suggest that hormonal signaling in dedifferentiated yew cells grown in vitro is different from that in plants and can be affected by the culture’s age. This might be a result of the high level of culture heterogeneity and constant auto-selection for intensive proliferation, which leads to the predominant formation of C14-OH taxoids versus C13-OH taxoids and a modified cell response to exogenous MeJ treatment.

## 1. Introduction

Taxane diterpenoids (taxoids), particularly paclitaxel, are plant secondary metabolites highly valued for their anti-cancer activities [1]. They are mostly obtained from different species of *Taxus* spp. (yew), where they are accumulated in small quantities [2]. Efforts have been made to produce taxoids in cell cultures as an alternative method to harvesting wild plants; however, there are only a few successful examples of commercial paclitaxel production using cell suspensions [3,4]. The biotechnological approach to taxoid synthesis is hampered by the low content of the target compounds in cell cultures as well as the potential risks of synthesis shift in the course of long-term in vitro cultivation [5].

The problem of low metabolite content in cell cultures is frequently addressed by chemical elicitation, i.e., adding specific compounds (stress-signaling molecules or precursors) to culture medium at the latter stages of culture growth, which simulates the biosynthesis of secondary metabolites in cells [2,6,7]. One of the most widely used elicitors is methyl jasmonate (MeJ), a non-specific stress signaling molecule that has the ability to respond to various stresses, including herbivore and pathogen attacks [8]. The effects of MeJ and jasmonic acid in plants have been comprehensively studied [9,10,11]. However, MeJ’s mode of action might be different in in vitro-grown cell cultures due to their specifics: dedifferentiation, artificial growth conditions, and the absence of organismic control [12,13,14]. MeJ has been used for the elicitation of yew cell cultures since the 1990s [4,15,16]. However, there is evidence that the MeJ effect may be species- and even genotype-specific and vary depending on cell immobilization and culture conditions, possibly due to a cross-talk between MeJ and other signaling pathways [17,18,19].

We previously reported that cell cultures of various yew species, including *Taxus baccata*, *T. canadensis*, *T. wallichiana*, and two *T. × media* hybrids, were able to synthesize both C13-oxygenated/hydroxylated (C13-OH) taxoids (decinnamoyl-taxinine J, taxuspine F, etc.) and C14-OH taxoids (7β-hydroxy-taxuyunnanin C, sinenxane C, taxuyunnanine C, 2α,5α,9α,10β,14β-pentaacetoxy-4(20), 11-taxadiene, and yunnanxane) [20,21]. Moreover, while C13-oxygenated/hydroxylated taxoids were detected in callus cell cultures of *T. × media* [21], cell cultures maintained for several years produced mainly C14-OH taxoids [21,22]. These results are of significant interest for commercial-scale cultivation, which requires stable qualitative and quantitative content of the desired secondary metabolites. However, little is known about the potential change in taxoid composition in the cell cultures during continuous maintenance. Further, little or no information is available about the effect of MeJ treatment on long-term cultured plant cell lines, while such information has both scientific and practical importance. We hypothesized that the cell culture’s response to elicitation may change depending on culture age.

The aim of the present study was to investigate the effect of MeJ elicitation on growth and the ability to synthesize C13-hydrohylated and C14-hydrohylated taxoids of the same cell culture of *Taxus wallichiana* at different ages (1.5 and 6 years after induction) during cultivation in flasks and pilot-scale bioreactors.

## 2. Materials and Methods

### 2.1. Plant Material and Culture Conditions

The ell culture of *Taxus wallichiana* Zucc., line Tw-bbg/B5-NB-pvp, used in this study was induced in January 2016 from a plant growing in the Minsk Botanical Garden of the National Academy of Sciences (Minsk, Belarus). Suspension culture was obtained from callus culture in June 2016 [23]. This cell line was yellow-colored and composed of small cell aggregates and individual cells. The aggregates consisted of meristem-like and parenchyma-like cells, predominantly of round shape. Cell culture was maintained in 250-mL and 500-mL flasks filled with, respectively, 40 mL and 80 mL of liquid nutrient medium composed of B5 [24] mineral salts, 1.0 g/L polyvinylpyrrolidone (PVP), 0.5 mg/L nicotinic acid, 0.1 mg/L thiamine, and 0.1 mg/L pyridoxine vitamins, 2 mg/L α-naphthalene acetic acid (NAA), and 0.3 mg/L 6-benzylaminopurine (BAP). Cultures were grown on an orbital shaker (90 rpm) at 26 ± 0.5 °C and 75% relative air humidity in darkness. For subculturing, 1 mL or 2 mL of the packed volume of the cell suspension was transferred to a fresh medium every 28 days.

Bioreactor cultivation was performed in 20-L bubble-type glass bioreactors of our own design (Institute of Plant Physiology of RAS, Moscow, Russia) with a 15-L working volume and in a 75-L bioreactor Electrolux-El-75 (Sweden) with a 50-L working volume. All bioreactors were operated in a semi-continuous mode, which was maintained by collecting a portion of cell suspension and simultaneously feeding fresh medium into the bioreactor at the beginning of the stationary phase of each subcultivation cycle. Cultivation was performed at 26 ± 0.5 °C in darkness; air supplied varied from 0.1 to 1.0 v/vpm depending on the growth phase of the cell culture. The initial density of cell suspension for bioreactor cultivation was 2–3 g dry weight (DW) per L medium.

### 2.2. Elicitation of Young and Old Cultures with Methyl Jasmonate (MeJ)

Elicitation experiments were performed in two series: in 2017 (1.5 years after culture induction) and in 2021–2022 (5–6 years after culture induction).

The first experiments were performed between May and July 2017. By this time, the culture was relatively «young» and had passed through five subculture cycles of callus and twelve cycles of suspension culture. In flask culture, MeJ at a final concentration of 100 µM (the concentration was chosen based on literature data [15]) was added to cell culture on day 21 of cultivation (the end of the exponential growth phase). In 20-L bioreactors, MeJ at a final concentration of 100 µM was added in the second cycle of cultivation at the end of the exponential growth phase (48th day of cultivation, 18th day from the beginning of the subculture cycle). The stock solution of MeJ (Sigma, Burlington, MA, USA) was prepared using methanol.

In 2021–2022, the second series of experiments was performed to compare the effect of MeJ on «young» and «old» cell cultures. By this time, the cell culture was 6-years-old and had passed through more than 70 subcultivation cycles. In this series of experiments, cultivation was performed in flasks and in 20- and 75-L bioreactors. MeJ was on day 19 in flasks and on day 17 in a 75-L bioreactor.

The influence of MeJ on cell culture growth and the production of taxoid diterpenoids was assessed as described below.

### 2.3. Growth Assessment

To assess the growth and physiological state of cell cultures in flasks and bioreactors, fresh weight (FW) and DW of cell biomass and cell viability were recorded periodically during the cultivation cycle as described earlier [23,25]. For FW evaluation, 10–15 mL aliquots of cell suspension were pipetted on paper filters, and the culture medium was removed under vacuum. The cell biomass was washed three times with distilled water under vacuum and weighted. Dry weight was recorded after ai-drying of cell biomass to a constant weight at 60 °C.

Growth index, specific growth rate, doubling time, economic coefficient, and productivity were calculated as previously described [20,23,25].

Cell viability was determined by staining with 0.025% Evans blue [23,25] as the percentage of cell aggregates composed of colorless (living) cells. A minimum of 250 cell aggregates were examined in each of the three replicates.

### 2.4. Analysis of Taxoid Diterpenoids in Cell Biomass

Taxoid diterpenoids were analyzed in the dry cell biomass by the UPLC-ESI-MS. Sample preparation for qualitative and quantitative analysis of taxoids was carried out according to previously published methods [20,21,22].

UPLC-ESI-MS (structural identification of diterpenoids): The structural identification of the compounds was performed as previously described [20,21,22]. The C13- and C14-hydroxylated taxoids were identified based on comparison of their chromatographic behavior and mass spectra (positive ions) with the standard samples and the literature data [21,26,27,28], as well as the interpretation of their mass spectra [20,21,22]. The following commercial reference samples of C13-hydroxylated taxoids were used: 7-xylosyl-10-deacetyltaxol, 10-deacetyltaxol, taxusin (ChromaDex, Los Angeles, CA, USA), cephalomannine, baccatin III, 10-deacetyl baccatin III, paclitaxel (Sigma Aldrich, Burlington, MA, USA), 13-acetyl-9-dihydrobaccatin III, and taxinin M (TRC, North York, Toronto, ON, Canada).

UPLC-ESI-MS (quantitative analysis): The analysis was performed using an Agilent 1260 Infinity instrument (Agilent Technologies, Santa Clara, CA, USA) equipped with a mass-selective detector (6100, Agilent Technologies, Santa Clara, CA, USA). Column: Poroshell 120 EC-C18 (100 mm × 3 mm, 2.7 µm, Agilent, Santa Clara, CA, USA). The column temperature was set at 43 °C, the mobile phase flow rate was 0.5 mL/min, and the injection volume was 0.5 µL. A 0.05% (*v*/*v*) solution of formic acid in water (solvent A) and acetonitrile (solvent B) was used as the mobile phase. Chromatographic separation was carried out in gradient elution mode. During the analysis, the composition of the mobile phase changed as follows (B, % by volume): 0–1 min—41%, 1–3 min—41→55%, 3–11 min—55%, 11–13 min—55→85%, and 13–17 min—85%. The analysis was performed in the positive ion detection mode (m/z range 100–1300, fragmentor 70). Ionization source parameters were the following: quadrupole temperature 100 °C, carrier gas (nitrogen) temperature 250 °C, nitrogen supply rate (spraying gas) 13 L/min, nitrogen pressure 2484 Torr, capillary voltage 4.0 kV. Quantitative determination of the content of individual taxoids was carried out by external calibration against standard samples of paclitaxel (Sigma, Burlington, MA, USA), 2α,5α,9α,10β,14β-pentaacetoxy-4(20), 11-taxadiene (previously isolated in our laboratory [20]), or taxusin (ChromaDex, Los Angeles, CA, USA). Under the described analytical conditions, the relative standard deviation of the taxoid retention times did not exceed 1%. In the working concentration range (5.5–277.7 µg/mL, 3.6–71.4 µg/mL, and 0.7–72.2 µg/mL for paclitaxel, 2α,5α,9α,10β,14β-pentaacetoxy-4(20), 11-taxadiene, and taxusin, respectively), the taxoid calibration curves were approximated by straight lines with coefficients of determination (*R*^2^) above 0.98. The relative standard deviation of taxoid peak areas did not exceed 10%.

### 2.5. Statistical Analysis

Data on growth assessment and the analysis of taxoids are presented as mean values with standard deviations recorded for the triplicates (three flasks or three fixed-size samples of cell suspension collected from bioreactors) for each data point. STATISTICA10 software (StatSoft©, Moscow, Russia) was used for processing the data.

## 3. Results

### 3.1. Effect of MeJ on the Growth and Biosynthetic Characteristics of the «Young» (1.5-Year-Old) Suspension Cell Culture of T. wallichiana in Flasks and Bioreactors

#### 3.1.1. Effect of MeJ on the Growth of the «Young» Suspension Cell Culture in Flasks and Bioreactors

**Figure 1:**
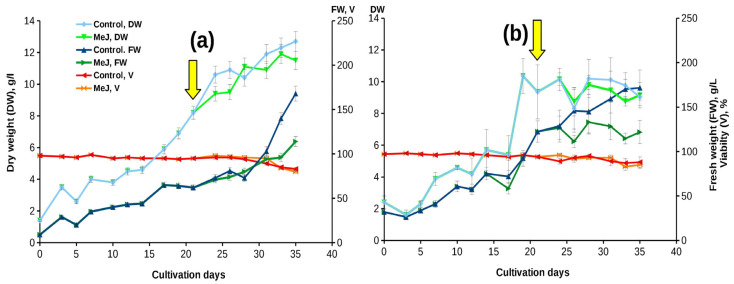
Growth curves of *Taxus wallichiana* suspension cell culture in flasks with and without MeJ elicitation: (**a**) the «young» culture (experiment performed in 2017); (**b**) the «old» cultures (experiment performed in 2021). The legend is the same as on (**a**). The arrow indicates the timepoint of MeJ addition (final concentration 100 µM) to cell suspension. MeJ—culture elicited with methyl jasmonate; DW—dry weight (g/L); FW—fresh weight (g/L); V—viability (%).

Growth curves and main growth parameters of «young» suspension cell culture of *T. wallichiana* in flasks are shown in Figure 1a and Table 1. The culture demonstrated rapid growth without a noticeable lag phase. The growth curve had a standard S-shape with a typical difference between FW and DW at the end of the subcultivation cycle, indicating significant hydration of the cells. Cell viability remained high (95–97%) during 28 days of cultivation and decreased to 82–85% by the end of the subculture cycle (Figure 1a). In addition, during the subcultivation cycle, minor step-like fluctuations in both fresh and dry weights were recorded (Figure 1a).

The addition of MeJ at a final concentration of 100 µM had almost no effect on the growth characteristics and viability of the cell culture (Figure 1a), but resulted in a significant (almost 1.5-fold) reduction of cell hydration by the end of the subcultivation cycle.

**Figure 2:**
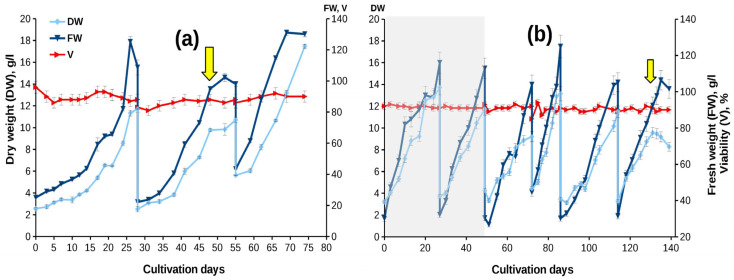
Growth curves of a suspension culture of *Taxus wallichiana* suspension cell culture: (**a**) the «young» culture in a 20-L bioreactor (experiment performed in 2017); (**b**) the «old» cultures in a 20-L bioreactor (first two cycles, gray background) and in a 75-L bioreactor (cycles 3–6) (experiment performed in 2021). 75-L bioreactors were inoculated from 20-L bioreactors. The legend is the same as on (**a**). The arrow indicates the timepoint of MeJ addition (final concentration 100 µM) to cell suspension. DW—dry weight (g/L); FW—fresh weight (g/L); V—viability (%).

Figure 2a and Table 2 present the growth curves and main growth parameters of the suspension cell culture of *T. wallichiana* grown in bioreactors.

The growth characteristics of the cell culture remained high during bioreactor cultivation. A minor decrease in the growth index recorded during bioreactor cultivation compared to flasks may be due to the high initial density of the cell culture. The addition of MeJ (final concentration 100 µM) had no effect on cell viability or maximum biomass accumulation during the same subculture cycle. Some increase in FW and DW accumulation was observed in the next subculture cycle following MeJ treatment. Interestingly, MeJ treatment slightly reduced cell hydration at the end of the next subcultivation cycle (cycle 3 in Figure 2a), as reflected by the lower difference between FW and DW.

#### 3.1.2. Effect of MeJ on Accumulation of Taxoid Diterpenoids in the «Young» Suspension Cell Culture in Flasks and Bioreactors

**Figure 3:**
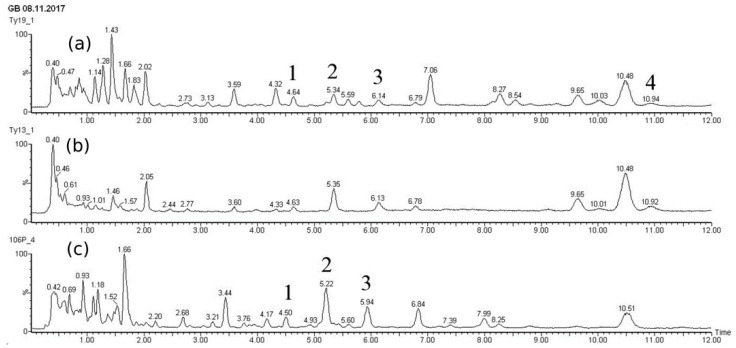
UPLC-ESI-MS chromatograms (total ion current, positive ion mode) of methanolic extracts from biomass of *Taxus wallichiana* cell suspension culture: (**a**) flasks, day 28, 7 days after MeJ elicitation (final concentration of 100 µM); (**b**) flasks, 28 days, control without elicitation; (**c**) 20-L bubble-type bioreactor, day 7 after MeJ elicitation. Peak numbers correspond to: 1—paclitaxel; 2—yunnanxane; 3—taxuyunnanine C; 4—sinenxan C. Axes: X—time, min; and Y—detector signal, relative intensity, %.

The UPLC-ESI-MS chromatograms of taxoid diterpenoids detected in the dry biomass of the «young» *T. wallichiana* cell suspension culture grown in flasks and bioreactors are presented in Figure 3 and Supplementary Figure S1. The MS spectra of the peaks corresponding to paclitaxel on UPLC-ESI-MS chromatograms are presented in Supplementary Figure S2.

The biomass of the suspension cell culture of *T. wallichiana* contained diterpenoids of the taxane series belonging to the structural type of taiwanxan (14-hydroxylated taxoids): sinenxane B, sinenxane C, taxuyunnanine C, 2α,5α,9α,10β,14β-pentaacetoxy-4(20), 11-taxadiene, yunnanxane, and an isomer of 7-hydroxy-2,5,10,14-tetra-acetoxy taxadiene. Under standard conditions in flasks, the dominant compounds were the C14-OH taxoids taxuyunnanine C and yunnanxane. C13-OH taxoids (paclitaxel) were also detected, but only in small amounts. The quantitative content of the main detected taxoids is shown in Table 3.

The addition of MeJ to the suspension cell culture caused a significant change in the composition of taxoids. As a result of MeJ action, paclitaxel content in cell biomass increased almost 10-fold, from 0.02 mg/gDW to 0.19 mg/gDW. Seven days after MeJ treatment (28–31 days of cultivation), the paclitaxel content reached almost 0.02% DW, which is comparable to its content in intact yew plants [29]. At the same time, the content of C14-OH taxoids (taxuyunnanine C and yunnanxane) changed insignificantly.

The results of taxoid analysis in the biomass of *T. wallichiana* cell suspension cultured in a 20-L bioreactor were comparable to those for flask culture (Figure 3, Table 3).

These results are in agreement with the literature [28,30,31] and suggest that cell culture of *T. wallichiana* grown in a bioreactor accumulated mainly C14-hydroxylated taxoids (taxuyunnanine C and yunnanxane), but their content was slightly lower compared to culture in flasks. The synthesis of paclitaxel in the cell culture was detected at the moment of adding MeJ (at the 48th day). At day 52, four days after adding MeJ, paclitaxel content reached 0.06 mg/gDW. Seven days after elicitation (at the 55th day of culturing), paclitaxel content was doubled at 0.11 mg/gDW, which was comparable to its level in intact *T. wallichiana* (Table 3). It is important that the paclitaxel content increase occurred in the next cycle of cultivation following elicitation and reached 0.15 mg/gDW by the end of the experiment.

The presence of C14-hydroxylated taxoids (taxuyunnanine C and yunnanxane), which were synthesized both before and after the addition of MeJ, was also recorded in the *T. wallichiana* cell culture grown in the bioreactor. The content of these compounds increased as a result of MeJ elicitation. For example, 7 days after elicitation (the 55th day of culture), the total content of C14-hydroxylated taxoids was 0.45 mg/gDW, while on day 77 of cultivation, it was 0.70 mg/gDW.

Our results confirm the predominant accumulation of C14-OH taxoids compared with other groups of taxoids in the suspension cell culture of *T. wallichiana*, regardless of the cultivation system (flask or bioreactor). However, the composition and ratio of individual taxoid compounds were different from those in plants. In particular, baccatin III (C13-OH taxoid) was not detected in *T. wallichiana* cell culture.

### 3.2. Effect of MeJ on the Growth and Byosynthetic Characteristics of the «Old» (6-Year-Old) Suspension Cell Culture of Taxus wallichiana in Flasks and Bioreactors

#### 3.2.1. Effect of MeJ on the Growth of the «Old» Suspension Cell Culture in Flasks and Bioreactors

When a 6-year-old suspension cell culture was grown in flasks and bioreactors, its growth characteristics resembled those recorded for the «young» culture (Figure 2, Table 2). A minor decrease in the growth index compared to «young» cultures may be due to a higher initial inoculum density. Interestingly, the culture preserved the step-wise growth curve observed for the «young» culture (Figure 2), but, due to the increase in the lag phase, the growth decrease was recorded at 2–5, 10–12, and 14–16 days of cultivation.

Similar to the experiments performed in 2017, the addition of MeJ (final concentration 100 µM) had little effect on culture growth characteristics in both flasks and bioreactors. Moreover, we observed the same effect of MeJ on reducing cellular hydration: in the control without elicitation, the ratio of FW to DW at the stationary growth stage was 1.4–1.5 times higher than in the variant with elicitation, which coincides with the results of the first experimental series performed in 2017 (Figure 2).

In 2021–2022, additional experiments were performed to upscale the cultivation of the suspension cell culture from 20-L to 75-L bioreactors. In these experiments, cell suspensions, after being cultured in 20-L bioreactors for two cycles, were inoculated into a 75-L bioreactor and grown for an additional four cycles of 14–28 days each (Figure 2a). The cell culture demonstrated good growth during all culture cycles in both 20-L and 75-L bioreactors. The growth characteristics were comparable to those recorded during cultivation in flasks and 20-L bioreactor cultivation of «young» cell culture in 2017 (Figure 2, Table 2). During the first three cultivation cycles in a 75-L bioreactor, the culture was adapting to the new cultivation conditions, so MeJ was added to the cell suspension in the fourth cultivation cycle on day 17, at the end of the exponential growth phase.

#### 3.2.2. Effect of MeJ on Accumulation of Taxoid Diterpenoids in the «Old» Suspension Cell Culture in Flasks and Bioreactors

**Figure 4:**
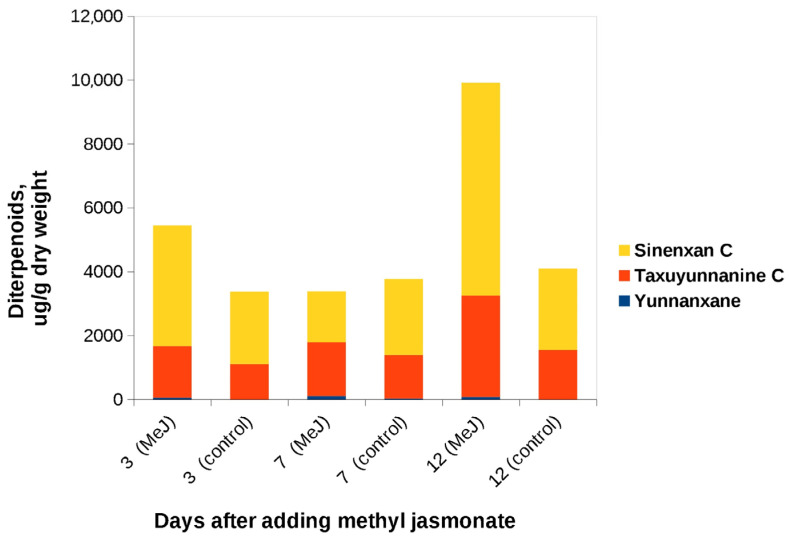
C14-hydroxylated taxoids content in cell biomass of the «old» suspension culture of *Taxus wallichiana* grown in flasks after addition of MeJ. Methyl jasmonate (final concentration of 100 µM) was added on the 19th day of cultivation. Sinenxan C, yunnanxane, and taxuyunnanine C contents were determined by the calibration curve for taxusin.

**Figure 5:**
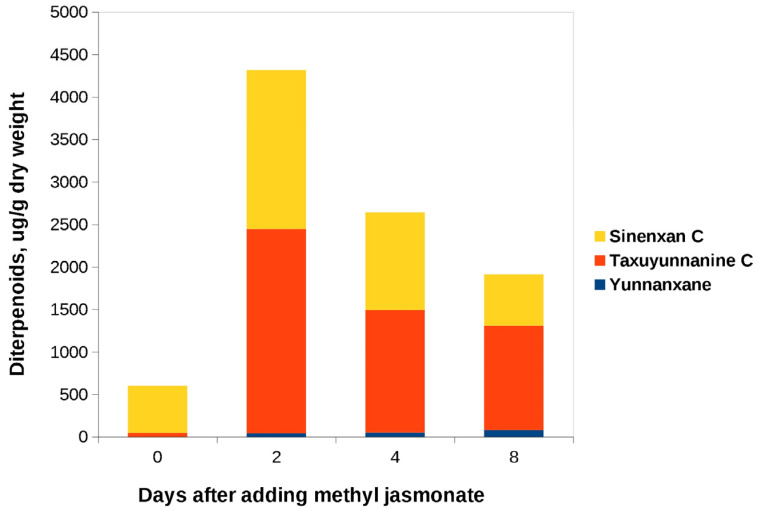
C14-hydroxylated taxoids content in cell biomass of the «old» suspension culture of *Taxus wallichiana* cultured in a 75-L bioreactor before (day 0) and after addition of MeJ. Methyl jasmonate (final concentration of 100 µM) was added on day 17 of culture cycle 4. Sinenxan C, yunnanxane, and taxuyunnanine C contents were determined by the calibration curve for taxusin.

MeJ was added to the «old» culture on day 19 of culture in flasks and on day 17 of bioreactor cultivation. In both flasks and bioreactors, C14-hydroxylated taxoids were found in significant amounts (Figure 4 and Figure 5). Synenxane C was the predominant compound regardless of the addition of MeJ; its content varied by more than 10-fold depending on cultivation system, from 0.55 mg/gDW (bioreactor, day 0, without elicitation) to 6.66 mg/gDW (flasks, day 12 after MeJ treatment). The content of yunnanxane ranged from zero to 0.1 mg/gDW. It is noteworthy that in the 2017 experiments, yunnanxane was quantitatively the predominant taxoid, with the content reaching 1 mg/gDW, while the “old” culture synthesized sinenxan C as the main compound. Elicitation with MeJ significantly increased yunnanxane content from 0–27 µg/g in the control culture to 40–100 µg/gDW in the culture with elicitation.

The effect of MeJ on the formation of C13-hydroxylated taxoids in the “old” culture was minor, and they were found in the cell biomass only in trace amounts. During cultivation in flasks, paclitaxel content on the 7th day after elicitation did not exceed 3.5 µg/gDW (Supplementary Figure S3). It is worth noting that, in addition to paclitaxel, other C13-hydroxylated taxoids appeared in trace amounts in the cell culture: 10-deacetyltaxol, 13-acetyl-9-dihydrobaccatin III, baccatin III, and cephalomannine. None of these compounds was detected in the control (not MeJ-treated) culture in flasks.

When cell suspension was cultured in a 75-L bioreactor, addition of MeJ resulted in the formation of trace amounts of C13-hydroxylated taxoids, but their composition was different from that in flasks. Interestingly, trace amounts (less than 3.5 µg/gDW) of the C13-OH compound, 10-deacetylbaccatin III, were found in culture on day 17 of culture cycle 4, that is, before the addition of MeJ, and remained until the end of the cultivation cycle. This compound was only found in a bioreactor culture. At the same time, paclitaxel and baccatin III were not found until the 14th day of cultivation after the addition of MeJ.

## 4. Discussion

Plant secondary metabolites perform a vital role in plant signaling and response to environmental conditions, including biotic and abiotic stresses, interactions with other plants, pollinators, and predators [9]. Taxoid diterpenoids are produced by a number of yew species as a part of their defense system against bark-colonizing fungi and oomycetes and are effective against the most widespread and important pathogens of conifers [32,33]. In medicine, these compounds are highly valued for their unique anti-tumor activities [3]. Although multiple studies have explored the use of yew cell culture to produce taxoids, these efforts are still hampered by the low content of the target compounds, particularly paclitaxel, in the cell cultures compared to plants [9].

Elicitation with MeJ is one of the most popular ways to stimulate the biosynthesis of the desired secondary metabolites in plant cell cultures. For example, it was effective in increasing the production of ecdysteroids in the cell cultures of *Ajuga turkestanica* [34] and ginsenosides in the cell cultures of *Panax notoginseng* [35]. In 1996, Yukimune et al. first reported that elicitation with MeJ improved the accumulation of paclitaxel and baccatin III (C13-OH taxoids) in *Taxus* spp. suspension cell cultures [15]. Since then, several *Taxus* species have been shown to be responsive to MeJ treatment. For example, MeJ induced the production of paclitaxel in the suspension cultures of *T. cuspidata* [36] and *T. canadensis* [17]. In *T. canadensis*, MeJ added to the culture medium increased paclitaxel production to 48.3 mg/L and baccatin III production to 53.6 mg/L compared to 0.4 mg/L of each of these compounds in control (non-elicited) cell culture [37]. In the work by Ketchum et al. [38], the greatest accumulation of paclitaxel in the cell cultures of *T. canadensis* and *T. cuspidata* occurred when MeJ was added to cultures at a final concentration of 200 μM on day 7 of the culture cycle. The concentration of paclitaxel increased in the extracellular (cell-free) medium to 23.4 mg/L per day within 7 days following elicitation. In cell culture of *T. baccata*, the addition of MeJ combined with lauryl alcohol stimulated a 2-fold increase in paclitaxel production without affecting culture growth characteristics [39].

Similarly, in the present study, production of C13-OH toxoids, including paclitaxel, in «young» cell cultures of *T. wallichiana* increased both in flasks and bioreactors in response to MeJ treatment, while culture growth and cell viability remained at a high level. Hence, the effect of MeJ on «young» cell culture of *T. wallichiana* was comparable to that in cell cultures of other yew species. The results are in agreement with the literature [28,30,31] and indirectly support the assumption of different regulation of the formation or accumulation of C13- and C14-hydroxylated taxoids in yew cells in vitro.

Other taxoids present in young cell cultures of *T. wallichiana* in this study were the C14-OH taxoids yunnanxane and taxuyunnanine C. Our earlier study revealed that cell cultures of different *Taxus* species and hybrids are able to produce C14-OH taxoids that accumulate in both cell biomass and culture medium [20].

It is noteworthy that nearly all studies referenced herein were performed using relatively young (recently induced) cell cultures. In this regard, it was interesting to compare taxoid synthesis and the effect of MeJ on the same cell culture after several years of in vitro maintenance by periodic subcultures.

The composition of taxoid diterpenoids in the cell culture of *T. wallichiana* notably changed after 6 years of cultivation. In the «old» culture, sinenxane C and taxuyunnanine C became predominant compounds, in contrast to «young» culture, which produced mostly yunnanxane and taxuyunnanine C in comparable amounts. In addition, the «old» cell culture reacted differently to MeJ treatment. By contrast to «young» cell culture, in the 6-year-old cell culture, MeJ increased the production of C14-OH taxoids sinenxane C and taxuyunnanine C but did not lead to the appearance of C13-OH paclitaxel. These results agree with the literature’s findings. For example, McKee et al. [19] reported that MeJ treatment of *T. cuspidata* and *T. canadiensis* cell lines producing paclitaxel led to an increase in the content of this compound. By contrast, MeJ could not induce paclitaxel synhesis in the cell lines that did not accumulate its UPLC-detectible levels before elicitation [19]. MeJ differently affected growth and taxoid (taxol, 10-deacetylbaccatin III, baccatin III, 10-deacetyltaxol, and cephalomannine) accumulation in cell culture of *T. globosa* depending on cell immobilization [39]. Interestingly, in the present study, MeJ induced the appearance of paclitaxel in the «young» culture but had only a minor effect on the content of C14-OH taxoids. By contrast, in the «old» culture, production of C14-OH taxoids was significantly affected by MeJ elicitation.

Several studies, including ours, demonstrated successful cultivation of taxoid-producing yew cell cultures in bioreactors of different volumes [3,21]. In the present study, the effect of MeJ was clearly related to culture age rather than a cultivation system (flasks or bioreactors). In both «young» and «old» cell suspensions, the responses to MeJ observed in bioreactor cultures resembled those in flasks. Therefore, flask cultivation could be effectively used for the initial screening as well as the elucidation of elicitation effects in yew cell cultures prior to scaling up the growth process to a more expensive bioreactor cultivation [20].

The response of the cell culture to treatment with plant growth regulators and elicitors may be different from that in plants due to the different physiology of plant cell culture as a population of undifferentiated cells lacking organismic control [13]. Our findings suggest that the response to MeJ treatment in the same cell culture depends on many factors and may change in the course of cultivation. Hormonal signaling in dedifferentiated yew cells grown in vitro is different from that in plants and changes with culture age. This might be a result of the high level of heterogeneity of cells in vitro and their constant auto-selection for proliferative intensity, which leads to the predominant formation of C14-OH taxoids versus C13-OH taxoids and a modified cell response to exogenous MeJ treatment. These observations may have important implications for the commercial production of both C14-OH and C13-OH taxoids, including paclitaxel, using cell culture technology. For example, some biotechnological companies, e.g., Phyton Biotech (https://phytonbiotech.com/, accessed 30 March 2023), claim on their website that they use cryopreservation in liquid nitrogen instead of periodic subcultures to ensure long-term preservation of the biosynthetic abilities of the productive cell lines.

## 5. Conclusions

*T. wallichiana* cell culture grown in flasks and bioreactors is a promising source of taxoids of different structural groups (C13-OH and C14-OH). Our results demonstrated that the composition of taxoids and cell responses to MeJ treatment depend on culture age rather than a cultivation system. «Young» cell culture can produce both C13-OH taxoids (including paclitaxel) and C14-OH taxoids. In addition, the content of C13-hydroxylated taxoids in a «young» cell culture may be significantly increased by elicitation with MeJ. By contrast, cell culture maintained in vitro for the long term (over 5 years, «old» culture) tends to produce predominantly C14-OH taxoids regardless of the cultivation system (flasks or bioreactors). MeJ treatment of the «old» culture stimulated accumulation of C14-OH but could not enhance biosynthesis of C13-OH taxoids. C14-hydroxylated taxoids can be successfully used in the treatment of various diseases, including metabolic syndromes and oncology. Hence, cell culture of *T. wallichiana* can be used as a source for different taxoid groups depending on culture age.

## Figures and Tables

**Table 1:** Growth parameters of the «young» and «old» *Taxus wallichiana* suspension cell cultures grown in flasks without elicitation.

	Growth Parameter					
	I	µ, day^−1^	τ, Days	M_max_, g/L	Y	P, g/(L × day)
	**«Young» culture** (experiment performed in 2017)					
Calculation based on FW	16.9	0.22	3.1	173.2	n/a	n/a
Calculation based on DW	10.2	0.21	3.3	12.7	0.33	0.39
Calculation based on the cell number	5.3	0.17	4.1	n/a	n/a	n/a
	**«Old» culture** (experiment performed in 2021)					
Calculation based on FW	4.4	0.27	2.6	168.2	n/a	n/a
Calculation based on DW	4.9	0.25	2.8	10.5	0.29	0.44

I—growth index; µ—specific growth rate; τ—doubling time; M_max_—maximum biomass accumulation; Y—economic coefficient; P—productivity; n/a—not applicable; FW—fresh weight; DW—dry weight.

**Table 2:** Growth parameters of the «young» and «old» *Taxus wallichiana* suspension cell cultures grown in 20-L or 75-L bioreactors, calculated based on dry weight.

Growth Cycle *	Growth Parameter					
	I	µ, day^−1^	τ, Days	M_max_, g/L	Y	P, g/(L × day)
	**«Young» culture, 20-L bioreactor** (experiment performed in 2017)					
1	4.6	0.26	2.7	11.7	0.32	0.32
2	4.3	0.21	3.3	10.5	0.30	0.30
3 (MeJ elicitation)	3.1	0.19	3.6	17.5	0.39	0.62
	**«Old» culture, 75-L bioreactor** (experiment performed in 2021)					
4	4.0	0.21	3.3	13.1	0.33	0.26
5	3.7	0.13	5.8	11.5	0.28	0.29
6 (MeJ elicitation)	3.2	0.16	4.9	9.47	0.21	0.28

* According to Figure 2a,b. I—growth index; µ—specific growth rate; τ—doubling time; M_max_—maximum biomass accumulation; Y—economic coefficient; P—productivity.

**Table 3:** Taxoid content in the biomass of the «young» suspension culture of *Taxus wallichiana* grown in flasks and a 20-L bioreactor (experiment performed in 2017).

Variant	Days of Culture	Taxoid Content, mg/gDW		
		Yunnanxane ^1^ (C14-OH Taxoid)	Taxuyunnanine C ^1^ (C14-OH Taxoid)	Paclitaxel (C13-OH Taxoid)
Flasks, control	28	0.77	0.53	0.02
	31	1.22	0.56	0.05
Flasks, MeJ	28	0.95	0.51	0.19
	31	1.02	0.65	0.18
20-L bioreactor, control	14	0.04	0.13	-
	28	0.12	0.02	-
	45	0.08	0.09	-
20-L bioreactor, MeJ (48 days of culture)	48	0.13	0.12	-
	52	0.15	0.13	0.06
	55	0.27	0.23	0.11
	77	0.36	0.34	0.15
Tree bark ^2^		-	-	0.13

^1^ Yunnanxane and taxuyunnanine C contents were determined by the calibration curves for taxusin or 2α,5α,9α,10β,14β-pentaacetoxy-4(20), 11-taxadiene. ^2^ Bark from the trunk of a *Taxus wallichiana* tree (Central Botanic Garden of the National Academy of Sciences of Belarus, Minsk).

## Data Availability

No new datasets were generated during this study.

## Supplementary Materials

The following supporting information can be downloaded at: https://www.mdpi.com/article/10.3390/biom13060969/s1, Figure S1: UPLC-ESI-MS chromatograms (extracted ion chromatograms for m/z 854.3 corresponding to [M+H]^+^ for paclitaxel) of methanolic extracts from the biomass of *Taxus wallichiana* suspension cell culture and solutions of standard samples of paclitaxel: (a) a sample of paclitexel isolated from the bark of *T. cuspidata*; (b) a methanolic extract from biomass of *T. wallichiana* «young» suspension cell culture, flasks, 28 days, control without elicitation; (c) a methanolic extract from biomass of *T. wallichiana* «young» suspension cell culture, flasks, day 28, 7 days after MeJ elicitation (final concentration 100 µM); (d) a paclitaxel standard sample purchased from Sigma (USA). Axes: X—time, min; Y—detector signal, relative intensity, %. Figure S2. MS spectra (positive ions) of the peaks corresponding to paclitaxel on UPLC-ESI-MS chromatograms of methanolic extracts from the biomass of *Taxus wallichiana* suspension cell culture and solutions of standard samples of paclitaxel in Figure S1: (a) a sample of paclitexel isolated from the bark of *T. cuspidata*; (b) a methanolic extract from biomass of *T. wallichiana* «young» suspension cell culture, flasks, 28 days, control without elicitation; (c) a methanolic extract from biomass of *T. wallichiana* «young» suspension cell culture, flasks, day 28, 7 days after MeJ elicitation (final concentration of 100 µM); (d) a paclitaxel standard sample purchased from Sigma (USA). Axes: X—m/z; Y—detector signal, relative intensity, %. Figure S3. UPLC-ESI-MS chromatograms (extracted ion chromatograms for m/z 876.3 corresponding to [M+Na]^+^ for paclitaxel) of methanolic extracts from the biomass of *Taxus wallichiana* «old» suspension cell culture and solution of paclitaxel standard sample: (a) a paclitaxel standard sample purchased from Sigma (USA); (b) a methanolic extract from biomass of *T. wallichiana* suspension cell culture, flasks, day 28, 7 days after MeJ elicitation (final concentration of 100 µM); (c) a methanolic extract from biomass of *T. wallichiana* suspension cell culture, flasks, day 28, control without elicitation. Axes: X—time, min; Y—detector signal, relative intensity, %.
