# Supplementary material for: Effect of Methyl Jasmonate on the Growth and Biosynthesis of C13- and C14-Hydroxylated Taxoids in the Cell Culture of Yew (Taxus wallichiana Zucc.) of Different Ages
Source: Biomolecules. 2023 Jun 9;13(6):969. doi: 10.3390/biom13060969 (PMC10296447; doi:10.3390/biom13060969)
Supplement: Supplementary file 1 [file biomolecules-13-00969-s001.zip › biomolecules-2394780-supplementary.pdf]

biomolecules

MDPI

Supplementary Materials

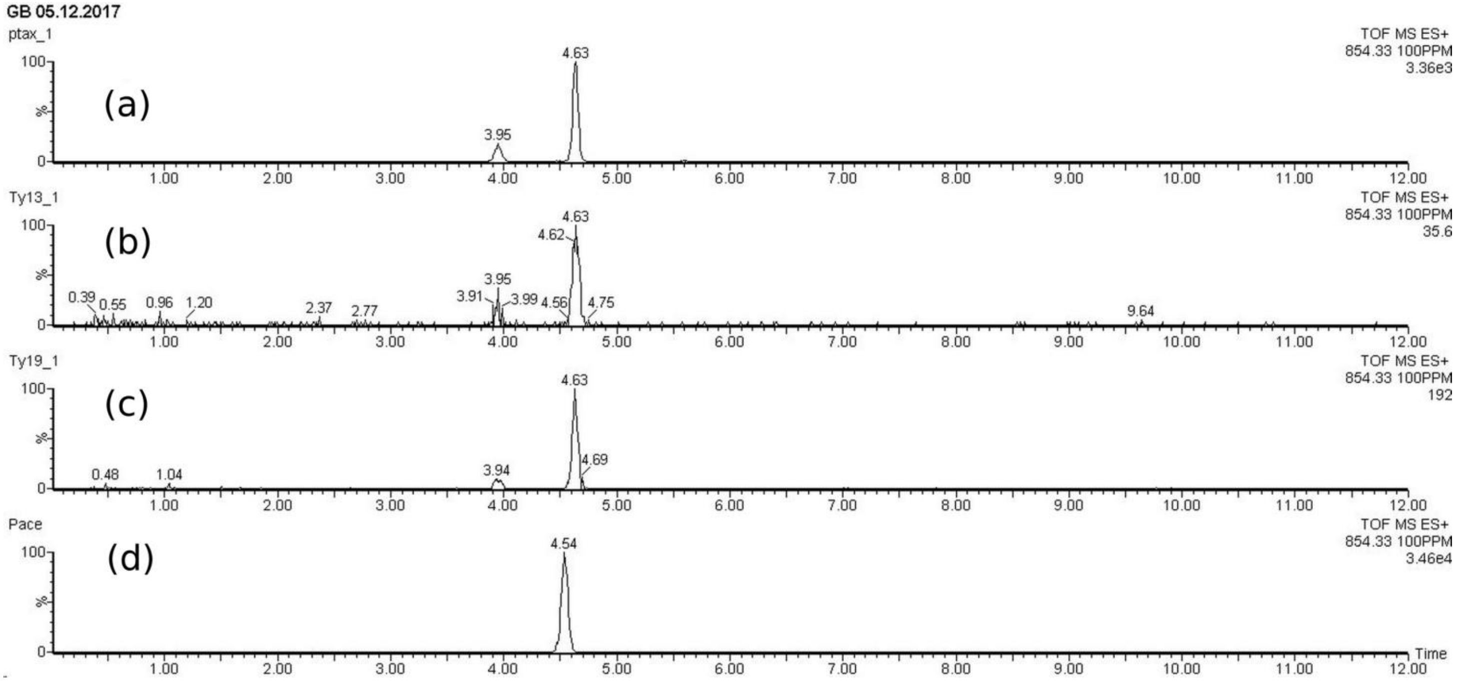

Fig. S1. UPLC-ESI-MS chromatograms (extracted ion chromatograms for m/z 854.3 corresponding to $[M+H]^+$ for paclitaxel) of methanolic extracts from biomass of *Taxus wallichiana* suspension cell culture and solutions of standard samples of paclitaxel: **(a)** – sample of paclitexel isolated from the bark of *T. cuspidata*; **(b)** – methanolic extract from biomass of *T. wallichiana* «young» suspension cell culture, flasks, 28 days, control without elicitation; **(c)** – methanolic extract from biomass of *T. wallichiana* «young» cell suspension culture, flasks, day 28, 7 days after MeJ elicitation (final concentration 100 μM); **(d)** – paclitaxel standard sample purchased from Sigma (USA). Axes: X – time, min; Y – detector signal, relative intensity, %.

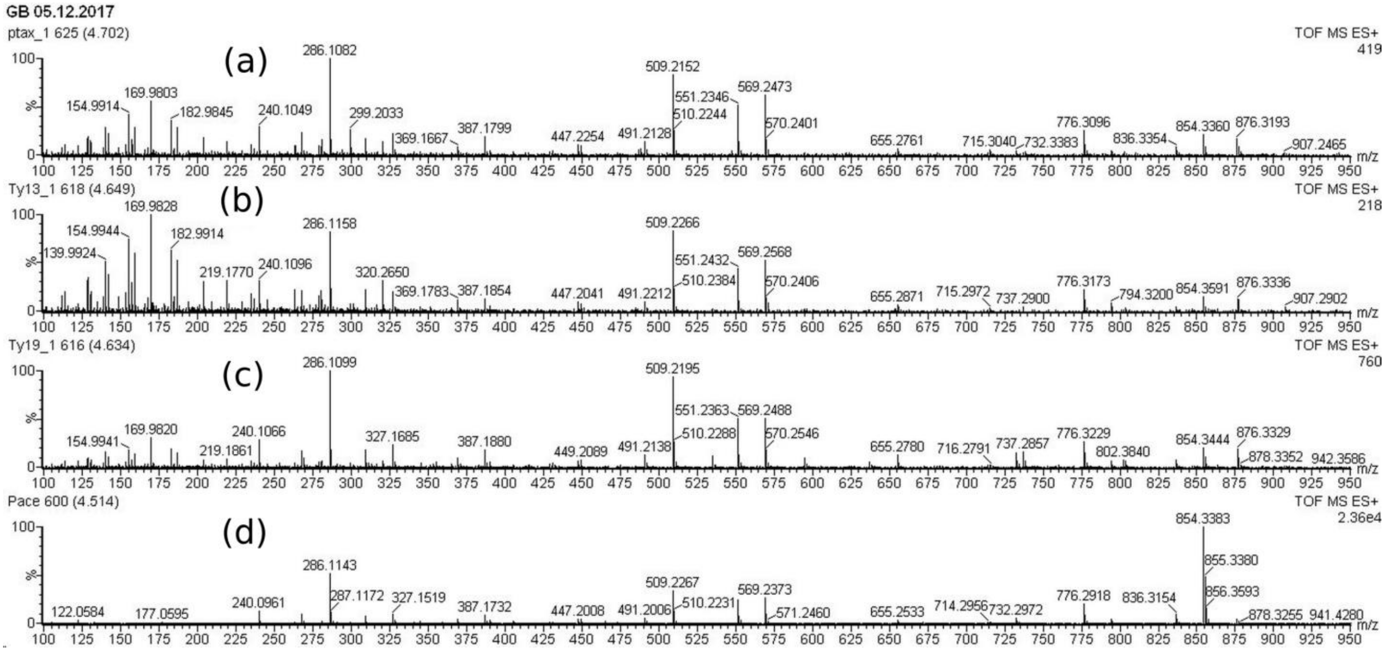

Fig S2. MS spectra (positive ions) of the peaks corresponding to paclitaxel on UPLC-ESI-MS chromatograms of methanolic extracts from biomass of *Taxus wallichiana* suspension cell culture and solutions of standard samples of paclitaxel in Fig. S1: **(a)** – sample of paclitexel isolated from the bark of *T. cuspidata*; **(b)** – methanolic extract from biomass of *T. wallichiana* «young» suspension cell culture, flasks, 28 days, control without elicitation; **(c)** – methanolic extract from biomass of *T. wallichiana* «young» suspension cell culture, flasks, day 28, 7 days after MeJ elicitation (final concentration100 μM); **(d)** – paclitaxel standard sample purchased from Sigma (USA). Axes: X – m/z; Y – detector signal, relative intensity, %.

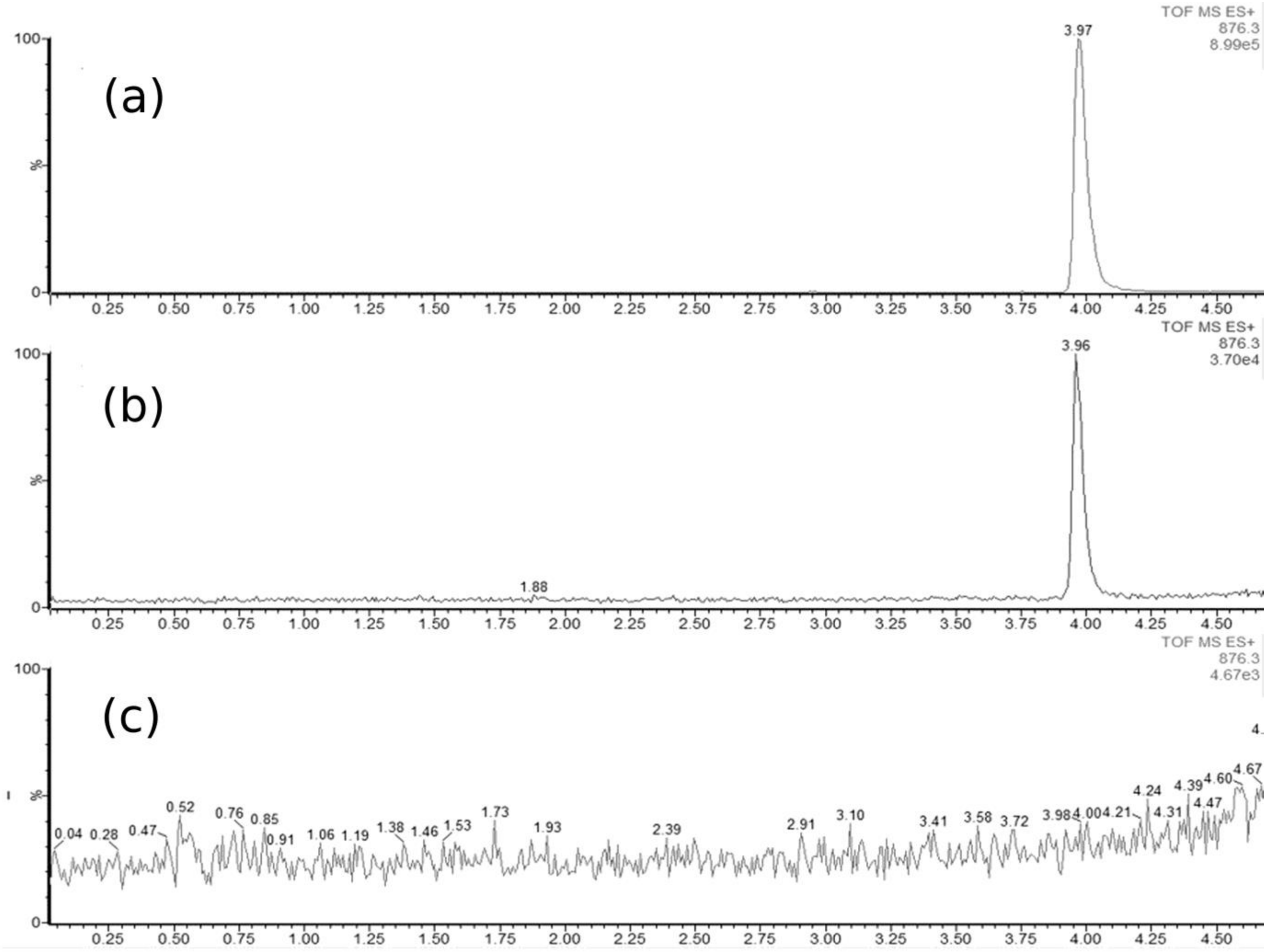

Fig. S3. UPLC-ESI-MS chromatograms (extracted ion chromatograms for m/z 876.3 corresponding to $[M+Na]^+$ for paclitaxel) of methanolic extracts from biomass of *Taxus wallichiana* «old» cell suspension culture and solution of paclitaxel standard sample: **(a)** – paclitaxel standard sample purchased from Sigma (USA); **(b)** – methanolic extract from biomass of *T. wallichiana* suspension cell culture, flasks, day 28, 7 days after MeJ elicitation (final concentration100 μM); **(c)** – methanolic extract from biomass of *T. wallichiana* cell suspension culture, flasks, day 28, control without elicitation. Axes: X – time, min; Y – detector signal, relative intensity, %.
